# Specific distribution of overexpressed aurora B kinase in interphase normal epithelial cells

**DOI:** 10.1186/1475-2867-5-31

**Published:** 2005-11-09

**Authors:** Ash-shafie Abdullah, Charlene Foong, Maki Murata-Hori

**Affiliations:** 1 Mammalian Cell Biology Group, Temasek Life Sciences Laboratory, 1 Research Link, National University of Singapore, 117604, Singapore; 2 Temasek Junior College, 22 Bedok South Road, 469278, Singapore

## Abstract

**Background:**

It is known that aurora B, a chromosomal passenger protein responsible for the proper progression of mitosis and cytokinesis, is overexpressed throughout the cell cycle in cancer cells. Overexpression of aurora B produced multinuclearity and induced aggressive metastasis, suggesting that overexpressed aurora B has multiple functions in cancer development. However, the detailed dynamics and functions of overexpressed aurora B are poorly understood.

**Results:**

We overexpressed GFP fused aurora B kinase in normal rat kidney epithelial cells. Using spinning disk confocal microscopy, we found that overexpressed aurora B-GFP was predominantly localized in the nucleus and along the cortex as a dot-like or short filamentous structure during interphase. Time-lapse imaging revealed that a cytoplasmic fraction of overexpressed aurora B-GFP was incorporated into the nucleus after cell division. Immunofluorescence studies showed that the nuclear fraction of overexpressed aurora B did not induce ectopic phosphorylation of histone H3 after cell division. The cytoplasmic fraction of overexpressed aurora B-GFP was mainly associated with cortical actin filaments but not stress fibers. Myosin II regulatory light chain, one of the possible targets for aurora B, did not colocalize with cortical aurora B-GFP, suggesting that overexpressed aurora B did not promote phosphorylation of myosin II regulatory light chain in interphase cells.

**Conclusion:**

We conclude that overexpressed aurora B has a specific localization pattern in interphase cells. Based on our findings, we propose that overexpressed aurora B targets the nuclear and cortical proteins during interphase, which may contribute to cancer development and tumor metastasis.

## Background

Aurora B kinase is a chromosomal passenger protein responsible for maintaining of chromosomal integrity through the proper coordination of mitosis and cytokinesis [1,2]. Regulation of aurora B kinase is such that expression levels of the protein peak at G2-M phase, while its kinase activity is maximal during mitosis [3]. Previous studies showed that aurora B kinase is involved in targeting and regulating the activity of a number of substrates, which in turn drive mitotic progression [1,2]. This is coupled by the fact that the expression of aurora B kinase is minimal during interphase, and the activity of the protein reaches its maximum just after the deactivation of CDK1 kinase [4], suggesting that the kinase activity of aurora B is mainly required during late mitosis. However, it has been found that in a number of cancer cell lines, aurora-B is constantly overexpressed throughout the cell cycle [5]. It has been shown that the overexpression of aurora B kinase in normal cells produced multinuclearity, perhaps leading to genetic instability, possibly due to defects in mitotic progression [5]. Moreover, the cells stably overexpressing aurora B showed more aggressive and malignant cancerous growth over control tumours [6]. These suggest that overexpressed aurora B kinase has multiple functions in cancer development. However, the mechanism by which overexpressed aurora B kinase promotes cancer development is poorly understood. In addition, although biochemical evidences revealed that aurora B is overexpressed throughout the cell cycle [5], little is known about the dynamics and functions of overexpressed aurora B during interphase. To examine if overexpressed aurora B has specific distribution throughout the cell cycle, we overexpressed aurora B-GFP in normal rat kidney epithelial (NRK) cells and analyzed the subcellular distribution of overexpressed aurora B using modern microscopic imaging. We found that overexpressed aurora B was preferentially associated with the nucleus and the cortex. Overexpression of aurora B induced neither ectopic phosphorylation of histone H3 nor excessive phosphorylation of myosin II regulatory light chain in interphase NRK cells, suggesting that aurora B has other specific targets in these regions.

## Results and Discussion

**Figure 1:**
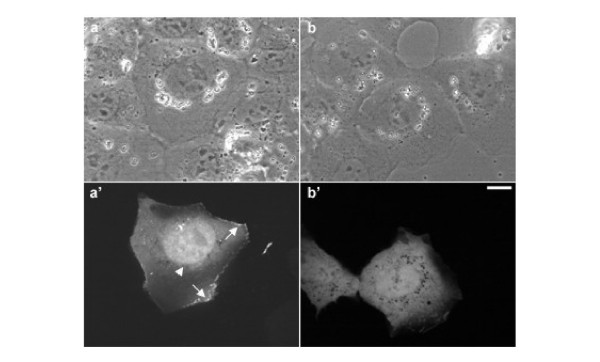
**Localization of overexpressed aurora B-GFP in an interphase NRK cell. **Spinning disk laser confocal microscopic images of living NRK cells overexpressing aurora B-GFP in interphase cells. The phase-contrast (a, b) and corresponding fluorescence images (a', b') show the distribution of overexpressed aurora B-GFP (a') or GFP alone (b'). Overexpressed aurora B-GFP is preferentially associated with nucleus (a', arrowhead) and the cortex (a', arrows), while GFP alone is diffusely distributed in the cell. Bar, 10 μm.

**Figure 2:**
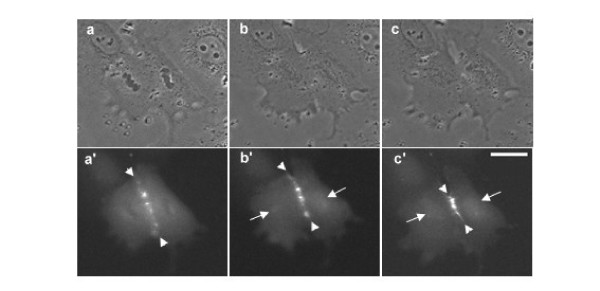
**Overexpressed aurora B is incorporated into the nucleus after cell division. **An NRK cell overexpressing aurora B-GFP was monitored by time-lapse microscopic imaging. Phase-contrast (a-c) and corresponding fluorescence images (a'-c') showed the dynamics of overexpressed aurora B-GFP during cytokinesis. Majority of aurora B is associated with the spindle midzone (a'-c', arrowheads) during cytokinesis. When the nuclear envelops started forming in daughter cells, a cytoplasmic fraction of overexpressed aurora B was incorporated into the nuclei (b' and c', arrows). Bar, 10 μm

**Figure 3:**
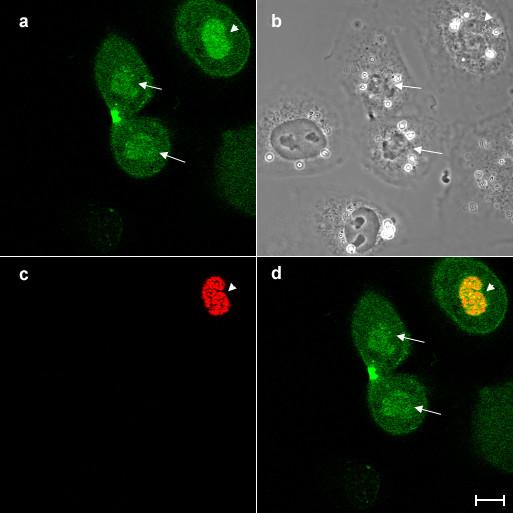
**Overexpression of aurora B kinase does not phosphorylate histone H3 in interphase NRK cells. **NRK cells overexpressing aurora B-GFP was stained with antibodies that specifically recognised histone H3 phosphorylated at Ser10 and then examined the expression of aurora B-GFP (a) and phosphorylation of histone H3 (c) by confocal laser microscopy. Corresponding phase and merged images (green; aurora B-GFP, red; phosphorylated histone H3) are shown in panels b and d, respectively. Although a fraction of aurora B-GFP is accumulated in the nucleus in a late telophase cell overexpressing aurora B-GFP, (arrows), phosphorylated histone H3 was not detected in the cell (c, d). Phosphorylated histone H3 was observed in a neighbouring prophase cell (arrowheads). Bar, 10 μm.

In order to investigate the distribution of overexpressed aurora B in interphase cells, NRK cells were transfected with GFP-tagged aurora B kinase [7] and were observed using spinning disk laser confocal microscope (Figure 1). Overexpressed aurora B-GFP was dispersedly localized in the nucleus (Figure 1a', arrowhead) but not nucleolus and associated with the cortex (Figure 1a', arrows), while GFP alone was diffusely localized in the cell and did not show any particular localization pattern (Figure 1b'). GFP tagged kinase-dead mutant of aurora B showed the similar localization pattern, indicating that overexpressed aurora B is distributed in the nuclei and along the cortex independently of its kinase activity (data not shown). It has been shown that aurora B was detected on the condensed chromosomes when the cells enter into prophase [7-10]. However, the localization pattern of overexpressed aurora B in the nucleus we observed here (Figure 1a', arrowhead) appeared to be different from that was observed in prophase cells [7]. Previous reports showed that aurora B localized to the centromeres as early as prophase and relocated to the spindle midzone during anaphase [7-9]. However, it is unknown if overexpressed aurora B has a unique localization pattern during cytokinesis. Thus, to understand the dynamics of overexpressed aurora B during cytokinesis, time-lapse imaging of dividing cells overexpressing aurora B-GFP was performed. A major fraction of aurora B-GFP was associated with the spindle midzone (Figure 2, arrowheads) [7]. Notably, as the chromosomes started decondensing, an increasing fluorescence signal of aurora B-GFP was observed around the chromosomes (Figure 2b', arrows). At late cytokinesis, the strong spherical mass of fluorescence signals was observed in the nuclear region (Figure 2c', arrows), suggesting that a cytoplasmic fraction of overexpressed aurora B enters the nucleus during nuclear envelope formation. Since aurora B phosphorylates histone H3 at Ser10 to catalyze chromosome condensation [8-10], we speculate that overexpressed aurora B may induce phosphorylation of histone H3 even after cell division. To test this possibility, we stained the cells overexpressing aurora B-GFP with antibodies that specifically recognize histone H3 phosphorylated at Ser10 (Figure 3). Phosphorylated histone H3 was observed in the neighboring mitotic cells (Figure 3c). However, we could not detect the phosphorylated histone H3 in the nucleus of G1 (late telophase) cells overexpressing aurora B (Figure 3, arrows), indicating that overexpressed aurora B did not induce ectopic phosphorylation of histone H3 in our experimental condition. A previous report showed that excessive phosphorylation of histone H3 by overexpressed aurora B appeared to be mainly induced during mitosis [6]. Based on these observations, we speculate that the nuclear fraction of overexpressed aurora B is likely to have other targets during interphase.

**Figure 4:**
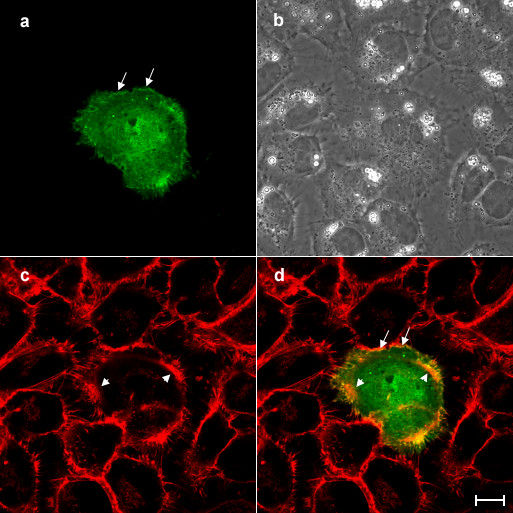
**Overexpressed aurora B is colocalized with cortical actin filaments but not stress fibers in interphase NRK cells. **An NRK cell overexpressing aurora B-GFP was stained with rhodamine-labelled phalloidin and then examined the subcelluar localization of aurora B-GFP (a) and actin filaments (c) by confocal laser microscopy. Corresponding phase and merged images (green; aurora B-GFP, red; actin filaments) are shown in panels b and d, respectively. Cortical aurora B-GFP is well colocalized with the short fragments of actin filaments around the cortex (a and d, arrows). However, overexpressed aurora B-GFP appeared not to colocalize with the thick stress actin filaments in the peripheral region of the cortical area (c and d, arrowheads). Bar, 10 μm.

**Figure 5:**
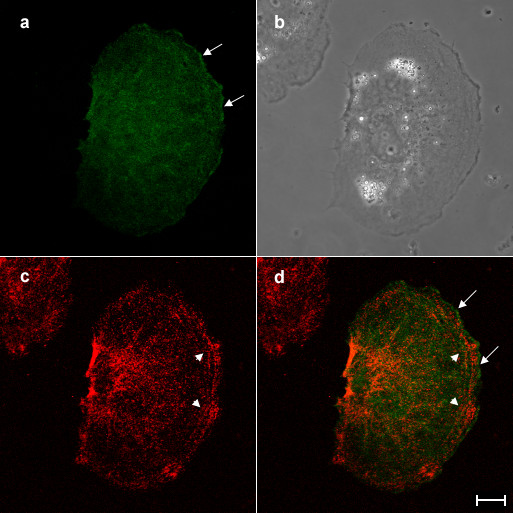
**Overexpressed aurora B is not colocalized with phosphorylated myosin II regulatory light chain in interphase NRK cells. **An NRK cell overexpressing aurora B-GFP was stained with antibodies that specifically recognised myosin II regulatory light chain phosphorylated at Ser19 and then examined the subcelluar localization of aurora B-GFP (a) and phopshorylated myosin II regulatory light chain (c) by confocal laser microscopy. Corresponding phase and merged images (green; aurora B-GFP, red; phosphorylated myosin II regulatory light chain) are shown in panels b and d, respectively. Overexpressed aurora B is associated with the cortex (a and d, arrows), while phosphorylated myosin II regulatory light chain is enriched in the cell periphery (c, and d, arrowheads). Bar, 10 μm.

The other fraction of overexpressed aurora B was preferentially distributed along the cortex with a dot-like or branched structure along the cortex in interphase cells, while GFP alone was uniformly localized in the cell (Figure 1). We tested if overexpressed aurora B colocalized with cortical actin filaments. The cells overexpressing aurora B-GFP were stained with rhodamine-labelled phalloidin and were then observed under the confocal microscope (Figure 4). Cortical aurora B-GFP was colocalized with short branched actin filaments along the membrane (Figure 4, arrows). Interestingly, overexpressed aurora B appeared not to be associated with the thick bundles of actin filaments (Figure 4, arrowheads). Since it has shown that aurora B kinase phosphorylates myosin II regulatory light chain at Ser19 [11], which stimulates actin-activated myosin II ATPase activity [12], we also stained the cells overexpressing aurora B-GFP with the antibodies that specifically recognize myosin II regulatory light chain phosphorylated at Ser19 (Figure 5). Consistently with the previous report [13], phosphorylated myosin II regulatory light chain was enriched around the cell periphery (Figure 5, arrowhead), while overexpressed aurora B-GFP was mainly associated with the cortex (Figure 5, arrow). Therefore, we hardly observed the colocalization of cortical aurora B-GFP with the phosphorylated myosin II regulatory light chain (Figure 5d). In addition, we did not detect an increase in the fluorescence signal of phosphorylated myosin II regulatory light chain in the cells overexpressing aurora B-GFP. We conclude that overexpressed aurora B does not promote phosphorylation of myosin II regulatory light chain in interphase NRK cells. During cytokinesis, aurora B is accumulated along not only midzone microtubules but also the lateral cortex through astral microtubules [14], indicating that aurora B may interact with cortical proteins which are associated with cortical actin filaments.

So far, several evidences have suggested that overexpression of aurora B promotes cancer development [5,6,15]. However, in our experimental condition, we did not observe any defects in cell division [7], cell morphology and cell growth. Since only a slight increase in the number of multinuclear cells was observe even when the normal fibroblast cells were transfected with a large amount of the plasmids encoding aurora B kinase [5], the expression level of aurora B in our experiments could be too weak to induce multinuclearity. Alternatively, overexpression of aurora B is not sufficient for the induction of carcinogenesis. Since the cells stably overexpressing aurora B were able to be isolated only when the p53 was mutated, it was suggested that overexpression of aurora B was induced after p53 defects in cancer development [6].

Our findings suggest that the targets for overexpressed aurora B are associated with the nucleus and the cortical actin filaments. The former target might be involved in the induction of polyploidy and/or signaling pathways in multi-step carcinogenesis, while the latter might be implicated in the development of metastasis. Our observations will help to search for the targets of overexpressed aurora B in mammalian cells.

## Materials and methods

### Cell Culture, Microscopy and Image Processing

Normal rat kidney epithelial cells (NRK-52E; American Type Culture Collection) were cultured in Kaighn's modified F12 medium supplemented with 10% fetal bovine serum, 100 U/ml Penicillin, and 100 μg/ml Streptomycin, on glass chamber dishes as previously described [16]. The cells were maintained at 37°C in an enclosed stage incubator built on top of an Axiovert 200 M inverted microscope (Carl Zeiss) and viewed with a 100×, numerical aperture 1.30, Oil Ph 3, Plan-NEOFLUAR lens, while another connected to a PerkinElmer RS-3 spinning disk confocal system. Live cell images were acquired with a cooled charge-coupled device camera (CoolSNAP_HQ_, Roper Scientific), processed with Metaview or a digital cooled ocra-ER camera (Hamamatsu). For fluorescence imaging using spinning disk confocal system, a CSU21 confocal optical scanner was used together with krypton-argon laser illumination source, with 488 nm excitation and emission filter (Chroma) HQ 525/50 M.

Fixed cells were viewed using inverted confocal Zeiss LSM 510 Meta microscope (Carl Zeiss) with a 100×, numerical aperture 1.25 Achroplan lens. Images were acquired using 488 nm Argon laser and 543 nm HeNe laser for excitation and signals were emitted through BP 505 – 530 nm filter and LP 560 nm filter.

### Transfection and Immunofluorescence

NRK cells were plated on a coverslip chamber dish and incubated for 18–24 h. Immediately before transfection, the cells were rinsed once F12K supplemented with 1% FBS or Opti-MEM I medium (Life Technologies). The cells were transfected with the DNA construct (2 μg) using Superfect or Effectene transfection reagent according to manufacturer's instructions (Qiagen).

For phosphorylated myosin II regulatory light chain immunofluorescence, cells were rinsed with warm cytoskeleton buffer [17] and fixed with 4% paraformaldehyde (EM Science) in warm cytoskeleton buffer for 10 min. They were then rinsed thoroughly using cytoskeleton buffer and permeabilized with 0.5% Triton X-100 incubated for 5 min. Fixed cells were rinsed with cytoskeleton buffer, blocked with 1% bovine serum albumin (BSA) (Roche Diagnostics) in PBS. Following on, the fixed cells were incubated with phospho-myosin light chain 2 (Ser19) polyclonal antibodies (Cell Signalling Technology) at a dilution of 1:100 in PBS with 1% BSA (PBS/BSA) for 45 min at 37°C. After thorough washing with PBS/BSA, cells were incubated with Alexa 546-conjugated goat anti mouse antibodies (Molecular Probes) at a dilution of 1:100 in PBS/BSA for 30 min at 37°C. For actin staining, the fixed cells were incubated with rhodamine labeled phalloidin (Molecular Probes) at a dilution of 1:50 in PBS for 30 min at 37°C.
